# Advanced MR techniques for the vessel wall: towards a better assessment of intracranial aneurysm instability

**DOI:** 10.1007/s00330-024-10583-0

**Published:** 2024-01-19

**Authors:** Shuang Zhai, Xinrui Wang

**Affiliations:** 1 Department of Outpatient, General Hospital of Northern Theatre Command, No.83 Wenhua Road, Shenhe District, Shenyang, 110016 China; 2 Department of Radiology, General Hospital of Northern Theatre Command, No.83, Wenhua Road, Shenhe District, Shenyang, 110016 China

Intracranial aneurysm (IA) is a common cerebrovascular disease with a prevalence of 3–5% in the general population [1], and it is more frequently detected with the technical improvements and broader availability of noninvasive neurovascular imaging. Clinicians are increasingly confronted with a dilemma regarding the therapeutic decision-making processes, namely, preventive intervention (surgical clipping or endovascular treatment) with inherent risks of complications, or conservative management with a relatively low lifetime rupture rate, whereas high morbidity and mortality associated with rupture [2]. Therefore, early recognition of unruptured intracranial aneurysms (UIAs) with high rupture risk is crucial, but it has long been challenging. Traditional risk evaluation based on patient’s characteristics and aneurysm morphology has limited sensitivity and specificity. Novel functional features of IAs demonstrated by advanced MRI technology may enhance risk stratification and improve patient management.

Visualizing the vessel wall is of great importance for intracranial vascular diseases, including aneurysms, since recent evidence suggests that UIAs may be structurally unstable before macroscopic growth on serial imaging [3]. High-resolution vessel wall imaging (HR-VWI) is an emerging imaging modality allowing for direct imaging of vessel wall abnormalities that complements conventional luminal imaging (i.e., CTA, MRA, and digital subtraction angiography (DSA)) in the evaluation of IAs [4]. In a recent survey of the American Society of Neuroradiology membership, 52.1% respondents indicated that their institution performed HR-VWI, and aneurysm characterization for rupture risk or culprit aneurysm (38.0%) was the third most common clinical indication [5].

Aneurysm wall enhancement (AWE) on post-contrast HR-VWI is the most studied characteristic and provides an appealing surrogate imaging marker for aneurysm instability (symptomatic, ruptured, or growing aneurysms). This enhancement is hypothesized to result from inflammation within the wall preceding and leading to the rupture, from a hemostatic thrombus at the rupture site, or from physical leakage of contrast medium from the aneurysm lumen secondary to endothelial disruption [6].

Although a reliable AWE assessment method is yet to be established, various approaches have been proposed to evaluate AWE patterns and signal intensity. Previous studies have qualitatively graded AWE patterns as none, focal, and circumferential enhancement. Focal enhancement was defined as enhancement involving a specific portion of the aneurysm like the neck, dome, or a bleb. Circumferential enhancement represented enhancement involving the entire aneurysm. Zhu et al showed that AWE pattern was the most useful parameter on contrast-enhanced VWI, indicating symptomatic status of UIAs independent of aneurysm size [7]. Edjlali et al further divided circumferential enhancement into thin (< 1 mm) and thick (> 1 mm) circumferential AWE, and found that the thick circumferential AWE demonstrated the highest specificity (84.4%) and negative predictive value (94.3%) for differentiating stable and unstable aneurysms [8].

In this issue of *European Radiology*, Fu et al studied a prospective cohort of 82 patients with 100 UIAs [9]. They quantitatively measured wall enhancement index (WEI) by comparing pre- and post-contrast signal intensity of aneurysm wall using normal brain parenchyma as reference, and showed that WEI was an independent risk factor for symptomatic aneurysms (OR = 3.31, *p* = 0.04). It is worth noting that most of the current grading methods for AWE have been performed on 2D multiplanar imaging and involved manual selection of regions of interest. As in Fu et al’s study, three IA wall slices (top, body, and bottom) were selected by the raters. However, the 2D approaches may limit the analysis of the entire aneurysm and hinder determining the risk of rupture. In a recent study, Raghuram et al proposed a new semi-automated pipeline for measuring AWE in three dimensions. They introduced three AWE metrics that describe different biological processes: 3D circumferential AWE (3D-CAWE) to quantify AWE for the entire aneurysm, aneurysm-specific contrast uptake (SAWE) to determine the average level of contrast uptake, and focal AWE (FAWE) to quantify focal AWE. Subgroup analysis of 68 aneurysms showed that the 3D-AWE method (AUC 0.67, sensitivity 53%, specificity 81%) outperformed the conventional 2D-AWE method (AUC = 0.48, sensitivity 13%, specificity 83%) in detecting symptomatic aneurysms [10]. Aneurysms with irregular morphology had increased enhancement, and blebs took up more contrast than the aneurysm sacs. 3D-AWE mapping could improve identification of these “high-risk” areas by visualizing AWE of the entire aneurysm, and thus enhance the process of aneurysm instability evaluation.

In their study, Fu et al also assessed aneurysmal wall permeability by calculating the volume transfer constant (K^trans^) on the dynamic contrast-enhanced (DCE) MRI co-registered to HR-VWI. K^trans^ (OR = 1.60, *p* = 0.04) was shown to be associated with aneurysm symptoms (sentinel headache or oculomotor nerve palsy) and positively correlated with WEI (Spearman’s coefficient of rank correlation = 0.41, *p* < 0.001) [9]. The rapid expansion and/or micro-perforations of the IAs are considered the pathogenesis of aneurysm symptoms, resulting in contrast leakage from the aneurysm into the adjacent region captured by time-resolved imaging. HR-VWI, along with a complementary imaging modality like DCE-MRI, will allow a better understanding of the aneurysm wall pathology in regard to wall inflammation and permeability and capture the precursor stage of IA instability.

Other MRI techniques for visualizing vessel walls, such as MRI-quantitative susceptibility mapping (QSM) and 7 T MRI, have also been applied in the assessment of IAs. MRI-QSM may be used as an objective tool for the detection of microbleeds related to sentinel headache, and identification of culprit IAs in patients with negative radiological evidence of subarachnoid hemorrhage or in patients with multiple aneurysms. 7 T HR-VWI has been shown to be a promising and powerful imaging technique for estimating aneurysm wall thickness, characterizing the triple-layered microstructure of aneurysmal wall, and detecting subtle wall enhancement otherwise unnoticed with 3 T scans [11].

In conclusion, evaluation of vessel walls by advanced MRI techniques is emerging as an important noninvasive approach to guide clinical management of IAs. On the one hand, more comprehensive and validated methods are still required to assess aneurysm wall characterization reliably in clinical practice. On the other hand, various imaging modalities can be used in combination to provide unique insights into the multifactorial process of aneurysm instability and improve the capabilities in determining IA rupture risk.

## Declarations

### Guarantor

The scientific guarantor of this publication is Xinrui Wang.

### Statistics and biometry

No complex statistical methods were necessary for this paper.

### Methodology

• commentary
